# Small workers are more persistent when providing and requiring help in a monomorphic ant

**DOI:** 10.1038/s41598-023-49012-5

**Published:** 2023-12-07

**Authors:** Filip Turza, Krzysztof Miler

**Affiliations:** 1 Doctoral School of Exact and Natural Sciences, Jagiellonian University, Prof. S. Łojasiewicza 11, 30-348 Kraków, Poland; 2 Institute of Environmental Sciences, Faculty of Biology, Jagiellonian University, Gronostajowa 7, 30-387 Kraków, Poland; 3 Institute of Systematics and Evolution of Animals, Polish Academy of Sciences, Sławkowska 17, 31-016 Kraków, Poland

**Keywords:** Animal behaviour, Entomology, Behavioural ecology

## Abstract

The common sand-dwelling *Formica cinerea* ants possess monomorphic workers, yet with considerable and easily identified size variation. Considering the importance of body size in polymorphic ants and other animals, we test whether size-dependent differences in behaviour occur in this species. We focus on the behaviour of large and small foragers in the context of rescue occurring between nestmates when one of them is entrapped and requires help. We show that workers of different sizes are characterized by a similar frequency of rescue activity and time delay to the first act of rescue. However, small workers rescue for longer than large workers. These results indicate that, although there is no size-related rescue specialization in *F. cinerea* foragers, small rescuers behave differently than large ones in terms of rescue persistence. Additionally, we show that small workers are more active when trapped. We suggest that variation in behavioural persistence of differently-sized workers may increase the efficiency of rescue actions. This study is the first to find a connection between body size and rescue behaviour in ants and the first to quantify and analyze the behaviour of individuals in need of rescue. These findings add substantially to our understanding of social insects and, more generally, highlight the need to study among-individual behavioural variation in social animals, including those in which body size is judged minute and irrelevant.

## Introduction

Rescue is a situation in which one individual provides help to another in danger^1^. In many animals including, e.g., bottlenose dolphins^2^, elephants^3^, rats^4^, some birds^5^, humpback whales^6^, mice^7^, wild boars^8^, and many species of primates^9–12^, rescue behaviour draws high scientific interest. It is especially well-researched and common in ants (reviewed in^13, 14^). Ants are considered to be organisms with a widespread rescue tendency, which might contribute to their ecological and evolutionary success^15, 16^. Belt^17^ first described army ants (*Eciton hamatum*) which tried to help their nestmates stuck under stones. Then, more detailed studies provided descriptions of rescue in ants trapped under the soil^18–20^, captured by predators such as antlions and spiders^1, 21^, or in confrontation with termites^22^. Rescue behaviour in ants can be studied both in the laboratory^23^ and in the natural setting^24^ using the same test that simulates natural rescue contexts in which one ant, the victim, is artificially trapped—tied by a thread to a piece of paper—and partially buried in the sand. When the victim is exposed to free nestmates, the latter often attempt to release the victim by pulling at its body, digging around it, excavating the sand, or even biting the thread entrapping it^23^. Although what the rescuers do in such situations is well quantified, the behaviour of the victims is largely neglected. These trapped individuals are considered passive during rescue attempts and dependent on their rescuers^25^.

There is a considerable, albeit largely unexplained variation in rescue behaviour expression between workers of the same colony^16, 26^. On the one hand, rescue behaviour increases colony fitness and so one might expect its high occurrence. For example, in harvester ants (*Veromesor pergandei*), the loss of 5 workers a day by spider predation translates to the loss of 65,700 seeds per year, which is a cost avoidable if nestmates show rescue behaviour towards endangered individuals^27^. In termite-eating ants (*Megaponera analis*), 32% of the injured ants die after confronting termites, but rescue behaviour minimizes these losses, allowing for up to 28.7% higher size of the colony and so mitigating the costs of loss of individuals^22^. On the other hand, rescue behaviour is considered risky for rescuing individuals^25, 28^, which might lower its occurrence. Therefore, some regulating processes connected to rescue behaviour expression are plausible. In general, little is known about these regulating processes^13^.

Body size is one of the most essential attributes of any living organism. It is tightly linked to many life-history traits and behavioural parameters^29^. It is also an organismal feature that is evolutionarily flexible and is one of the first to respond to natural selection^30, 31^. Research devoted to insect body size takes advantage of the fact that the exoskeleton is easier to measure than the soft bodies of other animals^32, 33^. Studying insects in this context greatly expands our knowledge about the importance of body size. In social insects, body size is indicated as a driver of the non-reproductive division of labour in workers of eusocial wasps ^34^, termites^35^, bumblebees^36^ and other bees^37^, as well as ants^38, 39^. Moreover, as shown by Trible and Kronauer^40^, accounting for body size is necessary to understand caste evolution and development among Formicidae.

In the so-called polymorphic ants with workers characterized by different sizes and shapes^41^, the relationship between morphology and task allocation is quite well studied^42–52^. Among ants, however, a majority of species are monomorphic^53^, i.e., without broad size or shape variation between workers^54^. These workers typically follow strong age polyethism and progress from performing one type of task to others as they age^41, 53^, but they might show some specialization or personality (consistent behavioural variation) towards different tasks in relation to their body size^55, 56^. These tendencies can be a result of factors influencing the development of larvae, such as nutrition or temperature (reviewed in^57^). Similarly, the relationship between body size and behaviour in monomorphic species is thought to increase the ergonomics of labour^58–64^. Factors such as the efficiency of energy expenditure or a decrease in mortality risks might shape task engagement in workers according to their body size^50, 51^.

Although size-dependent differences in behaviour seem to be common^43–47^ we have limited knowledge of their true adaptive significance, particularly within monomorphic ants^65^. We investigate, therefore, rescue behaviour variation among size-diversified workers that perform tasks outside the nest, the foragers. We use the monomorphic ant *Formica cinerea* as a model species because the evidence for inter-individual variation in the rescue behaviour of this species is well documented^66–72^ and because foragers of *F. cinerea* are visibly diverse in size^73, 74^. Our primary aim is to check whether foragers characterized by different body sizes differ in rescue behaviour directed towards nestmates. We propose a hypothesis that rescue activity would be higher when performed by small rather than large individuals. This is reasonable to expect in terms of risk management within the colony^75^. Specifically, larger workers can be more valuable than smaller workers because of different costs that need to be invested by the colony for the development of such workers in terms of biomass, which is difficult to compensate for when lost^76^. If so, small individuals should engage in risky rescue more than large individuals. Our alternative hypothesis is the reverse, i.e., that rescue activity would be higher in large rather than small workers. Rescue may be more efficient when performed by workers characterized by larger body sizes because of their higher rates of metabolism^77, 78^ or higher strength^79^. Considering that in workers of at least some polymorphic ants, larger workers live longer than smaller ones^80^, we measured this parameter among our foragers to exclude the possibility that our results would be related to life expectancy^68^. Indeed, life expectancy can play a role in rescue actions^66, 68, 72^. For instance, the ants with lower life expectancies seem to better discriminate which nestmates are worth rescuing^72^. Additionally, due to the general scarcity of data related to the behaviour of the victims, we present the first attempt at measuring and quantifying the behaviour of individuals in need of rescue. We explored whether the victim activity would depend on the size of the victim and rescue occurrence.

## Methods

The study was conducted in the field near Klucze (Błędowska Desert, Poland, 50°21′22″N 19°31′03″E) and the laboratory in Kraków (Institute of Environmental Sciences, Jagiellonian University, Poland). We used four independent colonies of *F. cinerea,* at least 500 m apart from each other. All studied colonies were polycalic, i.e., within which workers moved freely without aggression over various nests^74^. The colonies have been previously used in studies devoted to rescue behaviour^67, 70–72^.

### Behavioural experiment in the field

We performed four types of dyadic rescue behaviour tests, each with the victim classed as large (L) or small (S) and the potential rescuer also classed as large (L) or small (S).

The testing procedure was similar to that used in earlier studies^15, 24, 71^. For each test, a plastic ring (7 cm diameter × 7 cm high) was placed in an ant-free area near the nest entrance. The ring wall was coated with fluon (Sigma–Aldrich, Germany) to prevent nearby workers from entering the test area. Then, using clean forceps, a forager belonging to one of the size classes (L or S) was captured within a nearby area and inserted into a nylon loop attached to a piece of filter paper. Such a victim was placed inside the test area and partly covered with the surrounding sand. Immediately after placing a victim, a potential rescuer of a certain body size (L or S) was captured within a nearby area and placed in the test area. The ants were classified as large or small by eye in the field. Each ant, nylon thread, and filter paper were used only once. Each test lasted 5 min, started immediately after the placement of the victim and potential rescuer inside the ring, and was recorded using Sony HDR-CX625 cameras. After each test, forceps were sterilized in 98% ethanol to avoid direct transfer of cuticular hydrocarbons via the tested ants. All ants used in the tests were placed in plastic containers (separate for individuals categorized as large or small) and transported to the laboratory after the tests on the given day finished. The procedure was repeated 30 times for each dyad type (i.e., LL, LS, SL, SS) and colony (a total of 480 tests). We collected the data during an active foraging period of *F. cinerea*, i.e., between 9 AM and 6 PM^71, 81^. The order of the tests was counterbalanced by dyad type and colony, i.e. every day we performed tests for all four types of dyads for each of the four colonies. The order of the tested colonies and tests was changing daily (we performed 20 of all types of dyads per colony per day, i.e., 80 tests in total per day). The temperature during the six days over which the behavioural experiments in the field were performed ranged from 24–27 °C and humidity was between 52 and 65%. After each test day, all tested individuals were frozen and preserved in 98% ethanol until measured.

### Analysis of the recordings from the behavioural experiment

Each recording was coded (blinded) to prevent any potential observer bias and then analysed using the Behavioural Observation Research Interactive Software (BORIS;^82^). We noted whether in a given test any rescue behaviour such as digging around the entrapped individual, pulling at its body parts, transporting the sand covering it, and/or biting the thread entrapping it occurred at all, as well as the time to the first act of rescue and the total duration of rescue. This categorization of rescue behaviour was proposed by Nowbahari et al.^23^ and then subsequently used by multiple authors with similar dependent variables measured in each case, including the latency and total rescue duration^26, 67, 71, 83^. In addition, we analysed the behaviour of the ants in need of rescue. We measured the total duration of the victim’s activity including opening mandibles, kicking, and/or sand throwing. All behaviours were measured in seconds.

### Survival experiment in the laboratory

400 *F. cinerea* foragers were handpicked near the nest entrance from the four focal colonies (100 workers per colony) and transported to the laboratory. Ants during handpicking were divided into two classes: large (L) and small (S) workers (50 individuals per group, respectively). Similarly to e.g., Miler^66^, the ants were kept in plastic boxes (18 × 15 × 7 cm) separate for each colony and class, with fluon coating the inner walls (Sigma–Aldrich, Germany) to prevent escape, at a constant temperature of 24 °C, 40–60% relative humidity and a 12:12 day/night cycle. Ants were provided with water and 10% sucrose solution ad libitum. Ant mortality was checked daily from the moment of collection in the field until the end of the experiment, i.e., 200 days. Each day dead individuals were frozen and preserved in 98% ethanol until measured. The same was done with ants that remained alive after 200 days when the experiment was terminated.

### Body size measurements

To investigate the general distribution of size among foragers and confirm its unimodality in *F. cinerea*, we collected 400 active foragers (100 from each of the four focal colonies) and measured their body size. We also measured the body size of all frozen individuals that originated from the behavioural experiment in the field and survival experiment in the laboratory, in which the ants were categorized as large or small (1360 individuals in total, 960 workers from the behavioural experiment in the field and 400 workers from the survival experiment in the laboratory). We chose the maximum head width as a proxy of body size (a standard measurement of body size in ants, including *Formica* ants, e.g., ^84–86^) which was measured to the nearest 0.001 mm, using a digital microscope Delta Optical Smart 5MP PRO under 65 × magnification and Delta Optical Smart Analysis Pro software.

### Statistical analysis

All analyzes were performed using R^87^. To compare rescue occurrence (1—rescue, 0—no rescue), we used a generalized linear mixed model (lme4 package;^88^) with a binomial residual distribution, logit link function and included a random factor “colony” and a fixed factor “rescuer size” (L vs. S). Then, for further analysis, we used a subset of data that included only tests in which any rescue behaviour was observed. First, to compare the latency to the first rescue attempt, we used a generalized linear mixed model (lme4 package;^88^) with a gamma residual distribution, log link function and included a random factor “colony” and fixed factor “rescuer size” (L vs. S). Second, to compare the duration of rescue, we used a mixed-effects Cox proportional hazards model fit by maximum likelihood (coxme package;^89^) with a random factor “colony” and a fixed factor “rescuer size” (L vs. S). Data for tests during which rescue behaviour was interrupted at the end of the recording (i.e., 5 min) was censored to indicate that the behaviour of interest occurred beyond the ant's observation time (as in^72^). This was done to avoid biased outcomes within any group of workers due to some individuals that were still engaged in rescue after a recording period. Among a total number of 201 tests with any rescue, 81 were censored.

Furthermore, to compare the duration of rescue categories, we also used mixed-effects Cox proportional hazards models fit by maximum likelihood (coxme package;^89^) with a random factor “colony” and a fixed factor “rescuer size” (L vs. S). Data for tests during which a given rescue category was interrupted at the end of the recording (i.e., 5 min) was censored. Analogically as described above, 81 tests were censored for the same reasons as in the case of the total duration of rescue.

To compare the duration of activity in the victims, we used a mixed-effects Cox proportional hazards model fit by maximum likelihood (coxme package;^89^) with a random factor “colony” and the fixed factors of “victim size” (L vs. S) and “rescue occurrence” (0 vs. 1). We used a subset of data that included only tests in which any activity in the victim was observed. Similarly to the rescue duration analyses, data for tests during which victim activity was interrupted at the end of the recording (i.e., 5 min) was censored. Among a total number of 417 tests with any victim activity, 118 were censored.

To analyze mortality in large and small workers, we used a mixed-effects Cox proportional hazards model (coxme package;^89^) with a random factor “colony” and a fixed factor “group” (L vs. S). Data for ants that remained alive at the end of the survival experiment (i.e., after 200 days) was censored. Among a total number of 400 ants, 181 workers were censored.

To analyze body size distribution in each focal colony, we used a Shapiro–Wilk test of normality. To compare body size between workers classed as large and small in our two experiments, we used a linear mixed model (lme4 package;^88^) and two explanatory variables, a random factor “colony” and a fixed factor “group” (L vs. S).

### Ethical approval

No approval of research ethics committees was required to accomplish the goals of this study, because experimental work was conducted with an unregulated invertebrate species.

## Results

**Figure 1:**
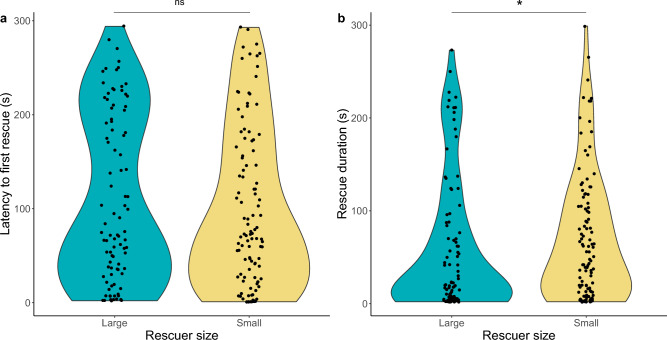
The latency to the first episode of rescue (**a**) and the duration of rescue (**b**) in differently-sized workers of *F. cinerea*. Dots represent each test with rescue occurrence. The violin plot outlines illustrate kernel probability density. Above plots, the “ns” indicates not significant (*p* > 0.05), whereas the asterisk indicates significant difference (*p* < 0.05) between groups. The latency to initiate the rescue episode showed no differences between tests. In terms of the duration of rescue behaviour, small workers demonstrated a longer time spent on rescue compared to large workers.

Rescue occurrence was similar between the two groups of workers (rescuer size: z = 1.58, *p* = 0.11; mean ± SE for small workers = 0.45 ± 0.03, mean ± SE for large workers = 0.38 ± 0.03). The latency to the first episode of rescue did not differ between tests (rescuer size: t_1,197_ = 0.77, *p* = 0.44; mean ± SE for small workers = 98.6 ± 8.09, mean ± SE for large workers = 110.6 ± 9.27; Fig. 1a). In terms of the duration of rescue behaviour, however, we found a significant effect of the rescuer size (z = − 2.36, *p* = 0.02; Fig. 1b), with small workers rescuing longer than large workers (mean ± SE for small workers = 70.40 ± 6.57, mean ± SE for large workers = 60.08 ± 7.58). In the analysis of rescue categories, differences were significant for the duration of digging (z = -3.26, p = 0.001) and sand transport (z = − 2.36, *p* = 0.02) but not pulling (z = − 1.89, *p* = 0.06) and thread biting (z = − 1.56, *p* = 0.12). In other words, small rescuers performed more digging and sand transport than large rescuers, which resulted in higher total durations of rescue in the former.

**Figure 2:**
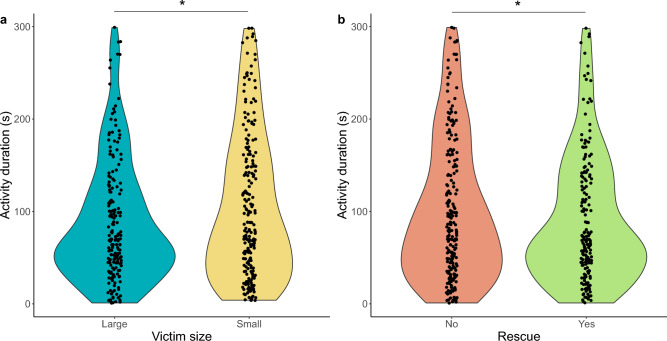
The duration of activity of the differently-sized victims of *F. cinerea* (**a**) and the duration of victims’ activity during rescue behaviour tests in which rescue occurred or not (**b**). Dots represent each test with activity occurrence. The violin plot outlines illustrate kernel probability density. Above plots, the asterisk indicates significant difference (*p* < 0.05) between groups. Small victims spent significantly more time being active than large victims. In terms of tests with and without rescue behaviour, victim activity was significantly lower in tests with rescue.

Small victims were characterized by higher activity than large victims (z = − 2.22, *p* = 0.03; Fig. 2a; mean ± SE for small workers = 103.59 ± 5.23, mean ± SD for large workers = 87.53 ± 4.67). In terms of tests with and without rescue behaviour, victim activity was lower in the former type of tests (z = 2.64, *p* = 0.01; Fig. 2b; mean ± SD for tests with rescue = 90.10 ± 5.05, mean ± SD for tests without rescue = 100.77 ± 4.94).

**Figure 3:**
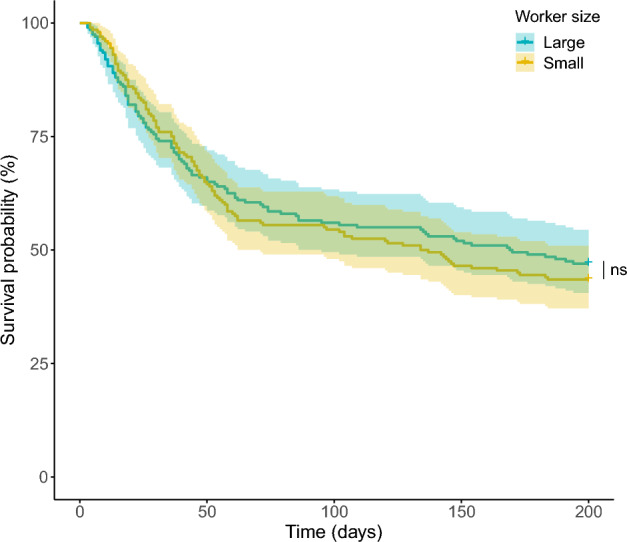
Survival curves for large and small foragers in *F. cinerea*. Shading indicates confidence intervals. There is no statistically significant difference between the groups (indicated by the “ns”).

Mortality did not differ between workers assigned to the two groups of workers: large vs. small (z = 0.48, *p* = 0.63, Fig. 3).

**Figure 4:**
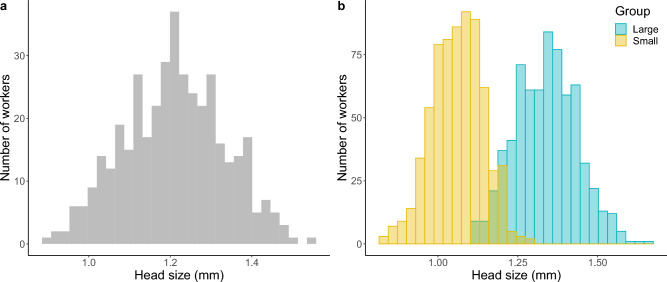
The unimodal distribution of head sizes among foragers (n = 400) of *F. cinerea* (**a**) and the distribution of head sizes among foragers targeted for our experiments and classed as large (n = 680) or small (n = 680) (**b**). The two distributions have significantly different means (small workers = 1.061 mm, large workers = 1.339 mm).

In *F. cinerea*, forager body size had a unimodal distribution (Fig. 4a). Shapiro–Wilk test of normality for each of the four colonies utilized in the study showed non-significant results and indicated that distributions did not deviate from normality (colony 1: W = 0.99, *p* = 0.58, colony 2: W = 0.99, *p* = 0.57, colony 3: W = 0.99, *p* = 0.48, colony 4: W = 0.98, *p* = 0.24). In turn, the results for the ants tested in our two experiments showed that there were significant size differences between workers classed as large or small (F_1,1355_ = 3354.40, *p* < 0.001; Fig. 4b). Although there was some overlap in size between classes, which indicated that our classification was not error-free, the vast majority of individuals used in the study were clearly larger or smaller and appropriately classed as such.

## Discussion

The present study offers the first investigation into the effect of the body size in a monomorphic (or any other) ant species in the context of rescue behaviour. Here we show a considerable size diversity in foragers in *F. cinerea*. As demonstrated by the results, these differently-sized individuals show no differences in longevity when measured in laboratory conditions. Furthermore, neither of our hypotheses regarding the effect of body size on rescue activity is confirmed. The decision to rescue a nestmate is not related to the body size of the rescuer, and this is also the case in terms of the time delay to the start of rescue activity. Small workers perform rescue for longer than large ones, but it seems likely that it is simply evidence of higher behavioural persistence. This difference between small and large workers stems from higher engagement in digging and sand transport in the former. It is also visible in the behaviour of workers during entrapment. Specifically, the activity of entrapped individuals is higher in small than large workers, which suggests higher persistence in attempts to self-release in the former. A recent study reported similar results in the highly polymorphic *Atta* leaf-cutting ants, in which smaller individuals are more persistent fighters, although the general probability of attack does not differ between workers of different sizes^90^. The authors suggest that smaller individuals might stimulate other individuals, including larger ones, to show aggression during colony defence. Somewhat increased rescue engagement which we describe here in small individuals may analogically function to facilitate nearby nestmates to join rescue action. Although this has not been explicitly studied, Nowbahari et al. already suggested that nestmates might facilitate rescue engagement^23^.

Differently sized workers engage in risky rescue acts with similar frequencies to those reported in previous research utilizing the same model species^66–71^. Of note, however, this is the first study to also measure, quantify, and report results concerning the activity of the victims. Their activity might indicate self-release attempts, as it causes physical disturbances which can remove whatever is entrapping them, and/or attract the attention of nestmates that might provide help. Activity is higher when the entrapped individual is small, which is reasonable considering that such individuals need more effort to remove whatever is ensnaring them and/or trigger rescue action. Nowbahari et al.^83^ reported that the use of motionless victims, anesthetized by chilling at − 4 °C for 2 min, triggers no rescue action compared to untreated active individuals. Thus, the activity of victims might be potentially used as an indicator of visual, chemical, and/or vibroacoustic cues to attract the attention of nestmates^1, 22, 23^. Curiously, in our study, victim activity decreases significantly in tests with rescue behaviour. This counterintuitive result, in our opinion, likely indicates that rescue is a cue for the victim to cease activity and wait. Future research needs to explore rescue from the perspective of the victims^13^. Indeed, the victim's behaviour may explain the rescuer's response and contribute to how well the rescue is performed, similarly to the social carrying behaviour in which the transported ant freezes to facilitate transport by another ant^38, 41, 91, 92^.

Regarding the behaviour of differently sized workers, there are some noteworthy results already reported in the literature in contexts other than rescue. For example, Herbers and Cunningham^55^ demonstrated that small workers in a monomorphic *Temnothorax longispinosus* engage in social interactions more frequently than large ones. Beshers and Traniello^59^ suggested that the most costly to produce and valuable (i.e., relatively large) individuals should be the least active. This is evident, for example, in the monomorphic *Myrmica kotokui*^93^, in which the largest individuals are the laziest ones. Large individuals of the monomorphic *Leptothorax acervorum* are also less aggressive than small ones^56^. How general such patterns of activity are in size-diversified workers of the monomorphic ant species remains to be determined. However, this requires a comprehensive approach that includes several measures of the behaviour, as in the current study and previous research devoted to polymorphic species^49–51^.

The workers in our study are arbitrarily classed into two size groups (large and small), the extremes of an unimodal distribution. Body size measurements and analysis confirmed that this method is accurate, however, it has some limitations. Such categorization is more appropriate for polymorphic ants, in which the discrete morphological castes arise from different development pathways (reviewed in^39^). Grouping monomorphic workers similarly to polymorphic workers might lead to misinterpretation of the results. Thus we suggest that in the case of monomorphic ants, treating the worker size as a continuous variable might be more appropriate. This, however, requires a different strategy of data collection than employed here in this study. In any case, in contrast to earlier findings, which mostly focus on size differences in workers performing different tasks^58–64^, our results show that in the monomorphic ant *F. cinerea,* body size diversity of workers may be observed even among the same task group, i.e., foragers. This highlights the importance of this body parameter, even in species with narrow and seemingly irrelevant worker size variation.

## Conclusions

Taken together, our data support a growing number of studies showing size-specific behavioural variation in animals^91, 94^. More specifically, we show that behavioural persistence in the context of rescue is higher in smaller sized workers of a monomorphic ant species. This research also introduces the measurement and analysis of the activity of ant workers in need of help. We suggest that future research on rescue behaviour should consider the poorly understood behaviour of entrapped individuals and its potential effect on rescuers.

## Data Availability

The datasets generated and analysed during the current study are available in the RODBUK repository, [https://doi.org/10.57903/UJ/RSAJWP].
